# In ovo delivery of carvacrol triggers expression of chemotactic factors, antimicrobial peptides and pro-inflammatory pathways in the yolk sac of broiler chicken embryos

**DOI:** 10.1186/s40104-024-01131-3

**Published:** 2025-01-19

**Authors:** Mila M. Y. Meijer, Henry van den Brand, Shahram Niknafs, Eugeni Roura

**Affiliations:** 1 Queensland Alliance for Agriculture and Food Innovation (QAAFI), The University of Queensland, Brisbane, Australia; 2 Department of Animal Sciences, Adaptation Physiology Group, Wageningen University and Research, Wageningen, the Netherlands

**Keywords:** Antimicrobial peptides, Broiler chicken, Carvacrol, Essential oils, Immunomodulation, In ovo, Yolk sac

## Abstract

**Background:**

Broiler chickens are most vulnerable immediately after hatching due to their immature immune systems, making them susceptible to infectious diseases. The yolk plays an important role in early immune defence by showing relevant antioxidant and passive immunity capabilities during broiler embryonic development. The immunomodulatory effects of phytogenic compound carvacrol have been widely reported. After in ovo delivery in the amniotic fluid during embryonic development carvacrol is known to migrate to the yolk sac. However, it is unknown whether carvacrol in the yolk could enhance defence responsiveness in the yolk sac. Therefore, the aim of this study was to improve early immune function in chicken embryos, and it was hypothesized that in ovo delivery of carvacrol would result in immunomodulatory effects in the yolk sac, potentially improving post-hatch resilience.

**Methods:**

On embryonic day (E)17.5, either a saline (control) or carvacrol solution was injected into the amniotic fluid. Yolk sac tissue samples were collected at E19.5, and transcriptomic analyses using RNA sequencing were performed, following functional enrichment analyses comparing the control (saline) and carvacrol-injected groups.

**Results:**

The results showed that 268 genes were upregulated and 174 downregulated in the carvacrol group compared to the control (*P* < 0.05; logFC < −0.5 or log FC > 0.5). Functional analyses of these differentially expressed genes, using KEGG, REACTOME, and Gene Ontology databases, showed enrichment of several immune-related pathways. This included the pathways ‘Antimicrobial peptides’ (*P* = 0.001) and ‘Chemoattractant activity’ (*P* = 0.004), amongst others. Moreover, the ‘NOD-like receptor signaling’ pathway was enriched (*P* = 0.002). Antimicrobial peptides are part of the innate immune defence and are amongst the molecules produced after the nucleotide oligomerization domain (NOD)-like receptor pathway activation. While these responses may be associated with an inflammatory reaction to an exogenous threat, they could also indicate that in ovo delivery of carvacrol could prepare the newly hatched chick against bacterial pathogens by potentially promoting antimicrobial peptide production through activation of NOD-like receptor signaling in the yolk sac.

**Conclusion:**

In conclusion, these findings suggest that in ovo delivery of carvacrol has the potential to enhance anti-pathogenic and pro-inflammatory responses in the yolk sac via upregulation of antimicrobial peptides, and NOD-like receptor pathways.

**Supplementary Information:**

The online version contains supplementary material available at 10.1186/s40104-024-01131-3.

## Background

Newly hatched broiler chickens are susceptible to infections partially because their immune system is still maturating [1, 2]. After hatching, adaptive immunity takes at least two weeks to develop, and in the meantime the chicken relies on maternal passive protection as well as innate immunity [3]. However, even the innate immune system is not properly matured in the first week post-hatching [2, 4]. This vulnerability can severely affect the broilers overall health and welfare, often leading to antibiotic treatments. Given the concern about the emergence of antimicrobial resistance, alternative strategies to aid in innate resistance against infections are required [5]. While often overlooked, the embryonic period poses the opportunity to improve immune function and disease protection prior to hatching. During embryonic development the yolk sac (YS) has two crucial roles, one as the main nutrient source and another as a temporary substitute for the developing immune organs [6]. The yolk is a source of maternal antibodies, which are transferred from the hen and aid in passive immunity [7]. However, the yolk sac tissue itself also plays a part in immune defense, by producing antimicrobial molecules, and more specifically avian beta-defensins (AvBD), throughout embryonic development [6, 8]. Because of these antimicrobial properties, the YS is a promising target for improving early immune functioning, thereby potentially reducing susceptibility to infections.

A widely recognized approach to intervene in the embryonic phase is by in ovo delivery. This technique involves injection of vaccines, nutrients, or other bio-active compounds into the amniotic fluid surrounding the developing embryo [9–12]. Plant derived compounds have gained interest as potential alternatives for antibiotics, because of their positive effects on gut health, which is closely linked to immune function [13]. Through the activity of the gut-associated lymphoid tissue, a healthy gut directly influences immune regulation and supports overall immunity. The monoterpenoid phenol carvacrol, more specifically, is known for its antimicrobial, anti-inflammatory and antioxidant properties [14–16]. Carvacrol, which is the main bioactive compound in essential oils such as oregano and thyme, also has been shown to reduce bacterial growth and downregulate inflammatory cytokines during a bacterial lipopolysaccharide challenge, thereby potentially reducing disease incidence [17, 18]. On the contrary, when applied in ovo, carvacrol has been shown to migrate and accumulate into the yolk of fertile broiler eggs and stimulated expression of pro-inflammatory cytokines at embryonic day (E)19.5 [19, 20]. However, it is unknown if the presence of carvacrol in the yolk may have any impact on immune mechanisms in the YS. Therefore, the aim of this study was to investigate the impact of in ovo delivery of carvacrol on YS immune defence mechanisms. Since immune function in the YS decreases towards hatching, E19.5 was chosen as target age [20]. Because of its antimicrobial and immune stimulating properties in ovo, it was hypothesized that in ovo delivery of carvacrol would stimulate antimicrobial defence pathways in the YS.

## Methods

This study adheres to the guidelines outlined in the Australian code for use of animals for scientific purposes as Animal Research: Reporting of in Vivo Experiments (ARRIVE) guidelines and was approved by The Animal Ethics Committee of the University of Queensland (Animal Ethics Unit, St Lucia, Queensland, Australia) under reference number 2019/AE000463.

### Experimental design

**Fig. 1:**
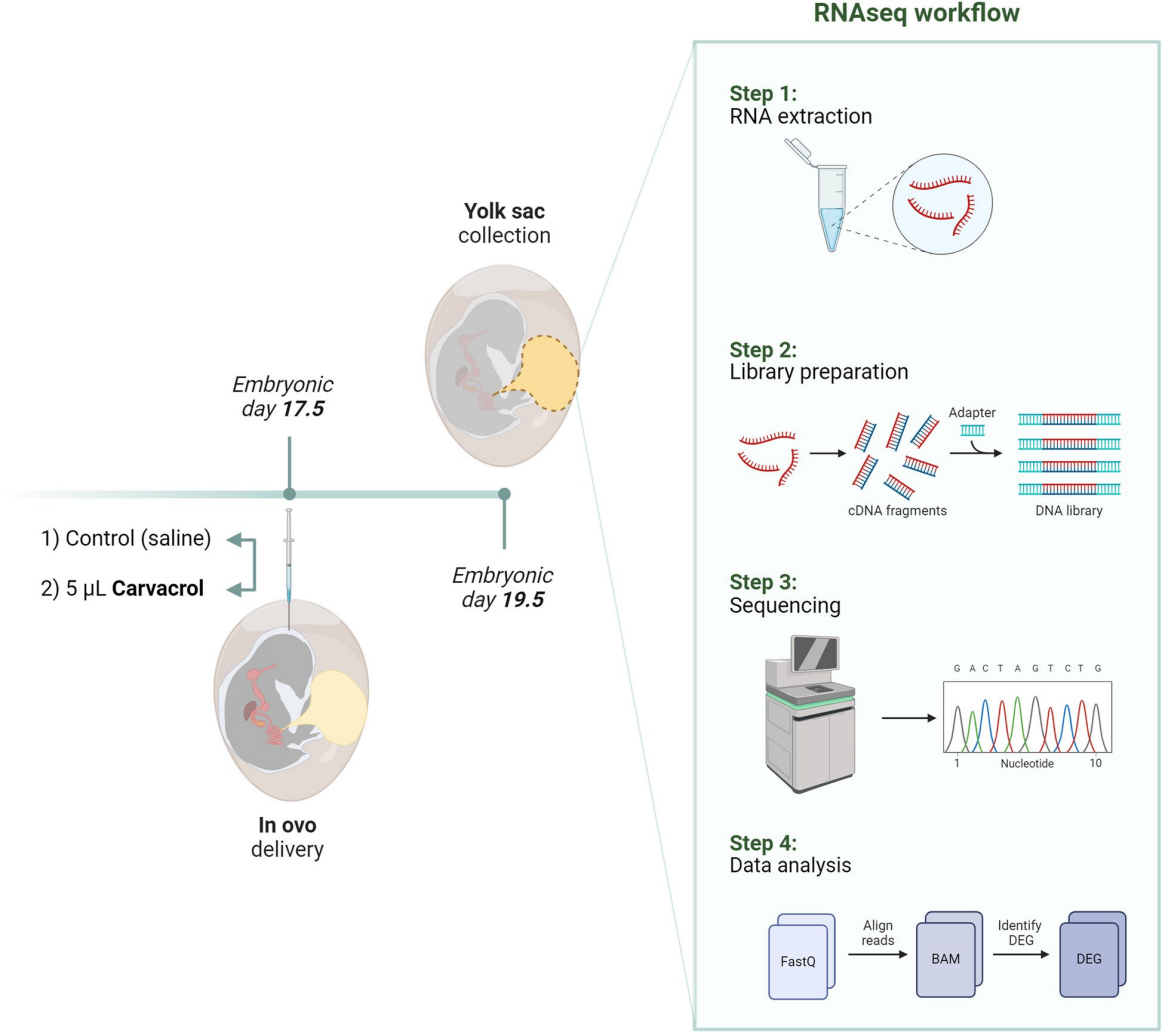
Experimental design. The experiment consisted of two in ovo delivery groups, a control group injected with saline, and a treatment group injected with 5 µL carvacrol. Solutions were injected into the amniotic fluid of fertile broiler eggs at embryonic day 17.5 and yolk sac (YS) tissue samples were collected at embryonic day 19.5. The YS transcriptomes of both treatments were sequenced using RNAseq and individual genes were compared. Created with BioRender.com

This experiment aimed to investigate effects of in ovo delivery of carvacrol in broiler breeder eggs on the YS transcriptome. The experiment consisted of two in ovo delivery groups, a control group injected with a 0.9% saline solution and a treatment group injected with 5 µL carvacrol in 0.9% saline solution. Solutions were injected into the amniotic fluid of fertile broiler eggs at embryonic day (E)17.5 and YS tissue samples were collected at E19.5. YS transcriptomes of both treatments (control; *n* = 5, carvacrol; *n* = 6) were sequenced using RNAseq and individual genes were compared (Fig. 1).

### Eggs, chickens, and housing

Fertile broiler eggs (Ross 308, *n* = 240) weighing on average 61 g (SD = 1.6 g) from a 38-week-old breeder flock, were supplied by a local commercial hatchery (Woodlands, Beerwah, QLD, Australia) and moved to the University of Queensland experimental chicken hatchery (St Lucia, Queensland, Australia). Eggs were incubated for 17.5 d in a setter (Ova-Easy 580 Advance Series II, Brinsea, FL, USA) with 6 levels and 2 trays per level, making a total of 12 trays. The incubator was set at a temperature of 37.8 °C, relative humidity of 57%, and a turning interval of 60 min over an angle of 90°. The moment the eggs were placed in the setter, which was already at the right temperature and relative humidity, was defined as the start of incubation. In ovo injection took place at E17.5 (see section below) and thereafter 48 eggs were (*n* = 24 per in ovo treatment) were placed back in the setter until tissue collection, while the other 192 were transferred to hatcher incubators for other experimental purposes (out of the scope of this paper).

### In ovo injection

Eggs were injected with either saline or carvacrol solution at E17.5. First, eggshells were sterilized with a 70% ethanol wipe. Secondly, a hole was drilled into the eggshell on the air cell location of the egg, using a multipurpose rotary tool (Ryobi EHT150, Ryobi, Hiroshima, Japan) with an arrow-shaped insert (Dremel High-Speed Cutter 6.4 mm, Dremel, Mount Prospect, IL, USA), while ensuring the membranes remained intact. A precision needle [23G 1 ¼ (32 mm)] was used to inject into the amniotic fluid. The hole caused by injection was sealed with a droplet of beeswax.

Eggs received injections of either 1 mL of 0.9% sterile saline solution (NaCl 0.9% in water, Baxter, Deerfield, IL, USA, CAS: 7647-14-5) or 5 µL of carvacrol (Sigma Aldrich, St. Louis, MO, USA, CAS: 499-75-2, purity 99%) dissolved in 1 mL of 0.9% sterile saline solution.

To prepare the carvacrol solution, 2 mL of carvacrol was combined with 2 mL of the non-ionic surfactant polysorbate 80 (Tween^®^80, Sigma Aldrich, CAS: 9005-65-6) for solubilization, as described by Niknafs et al. [21]. The mixture was gently pipetted for 1 min to ensure proper mixing. Subsequently, 2 mL of 0.9% sterile saline solution was added and, by pipetting, mixed with the carvacrol-polysorbate 80 solution. This addition and mixing of 2 mL saline was repeated three more times, bringing the total volume to 12 mL. Finally, the solution was adjusted to a total volume of 40 mL by adding 28 mL of sterile saline and shaking vigorously. This resulted in a 5% carvacrol stock solution, which was further used to prepare working solutions of 0.5% carvacrol.

### Tissue sampling and RNA extraction

Ten eggs per treatment were randomly selected at E19.5, the eggshell was removed, and embryos were euthanized immediately using decapitation. Thereafter, the YS was excised from the other tissues. Approximately 5 mm^3^ of tissue was collected from the middle of the YS. Samples were manually rinsed with PBS for 10 s, collected in RNAlater (Sigma Aldrich) and stored at −80 °C until analysis. Total RNA was isolated from the YS tissue, using RNeasy Universal kit (Qiagen, Hilden, Germany), following the manufacturer’s instructions. In brief, 50–100 mg of YS tissue was homogenized in Qiazol lysis reagent (Qiagen, Hilden, Germany), using a TissueRuptor (Qiagen, Hilden, Germany). Afterwards, gDNA eliminator solution (Qiagen, Hilden, Germany) was used to remove genomic DNA. Then, multiple washing steps were performed with chloroform, 70% ethanol and washing buffers, using Rneasy spin columns and a micro-centrifuge according to the manufacturer’s instructions. Purified RNA was diluted with RNase-free water and stored at −20 °C. Evaluation of RNA samples in terms of quality and quantity was conducted in an Agilent 2100 Bioanalyzer (Agilent Technologies, Santa Clara, USA). A quality threshold of RIN (RNA integrity number) > 7 was used to screen RNA samples for sequencing, which resulted in the selection of six samples per treatment (*n* = 6).

### Library preparation and RNA sequencing

Library preparation and RNA sequencing were performed by the Australian Genome Research Facility (AGRF Ltd., Melbourne, VIC, Australia). The analyses adhered to the specifications of ISO1702 as outlined by the National Association of Testing Authorities (NATA) of Australia. In short, 12 poly-A RNA stranded samples were sequenced, using the Illumina NovaSeq X Plus platform, producing an average of 4.9 million paired-end sequence reads per sample (average of 1.5 GB per sample). Pair-end reads were 150 bp in length. Primary sequence data was generated, using the Illumina DRAGEN BCL Convert 07.021.645.4.0.3 pipeline.

### Sequence reads quality control and mapping

The initial bioinformatics analysis included demultiplexing and quality control. The data was processed through an RNA-seq expression analysis workflow, which included steps, such as trimming, alignment, transcript assembly, feature quantification, and differential expression analysis. The sequence files were produced as standard FASTQ format. All 12 samples had good base sequence quality with > 95% bases above Q30 across all samples. The reads underwent screening to the presence of adapters, cross-species contamination, and overrepresented sequences. Following this, the high-quality sequence reads were aligned to the reference genome (*Gallus Gallus* GRCg7b), using STAR aligner (v2.5.3a), as per the manual (available at https://github.com/alexdobin/STAR). The resulting alignment files were generated in compressed BAM format. Read counts were then summarized based on uniquely mapped reads for each known gene, using the subread package of the featureCounts v1.5.3 utility for EdgeR. The StringTie tool v2.1.4 was utilized to assemble the transcripts, using the reads alignment and reference annotation-based assembly option (RABT). The RefSeq annotation, containing both coding and non-coding annotation for the genome, was used as a guide. On average 94.9% of the paired reads were successfully mapped to the reference genome, with an average of 77.2% uniquely annotated to single genes. Using MDS plots, one sample from the control treatment was determined as being very different from all others and was therefore removed from further analyses, leading to *n* = 5 for the control and *n* = 6 for the carvacrol injected group. The RNA-seq data was validated, using RT-qPCR analysis, the detailed method is provided as Supplementary methods (Additional file 1).

### Differentially expressed genes and functional analyses

The statistical analyses were conducted following the methodology Niknafs and co-workers [22]. In short, the differential expression analysis was performed in EdgeR (version 3.38.4), using R package (4.2.2). EdgeR is a tool designed for the identification and quantification of differential expression in RNAseq data, relying on the counts of uniquely mapped reads for each gene of *Gallus gallus*. To normalize sample counts EdgeR’s default normalization method was used. Differential expression between treatment groups was quantified, using a general linear model (GLM), and differentially expressed genes (DEG) were determined based on a threshold of *P* < 0.05 and log fold change (logFC) < −0.5 or logFC > 0.5. For metabolic pathway enrichment analyses, the DEG between the 2 groups (saline injected control versus carvacrol injected group) were used as input. Enrichment and functional analyses (functional annotation clustering) were performed in DAVID (Database for Annotation, Visualization, and Integrated Discovery), with a significance threshold set at *P* < 0.05 [23]. Enriched metabolic pathways and terms across the databases Kyoto Encyclopedia of Genes and Genomes (KEGG), Gene Ontology (GO), and REACTOME were integrated to comprehend the functional implications of the DEG identified between the two treatments.

## Results

**Fig. 2:**
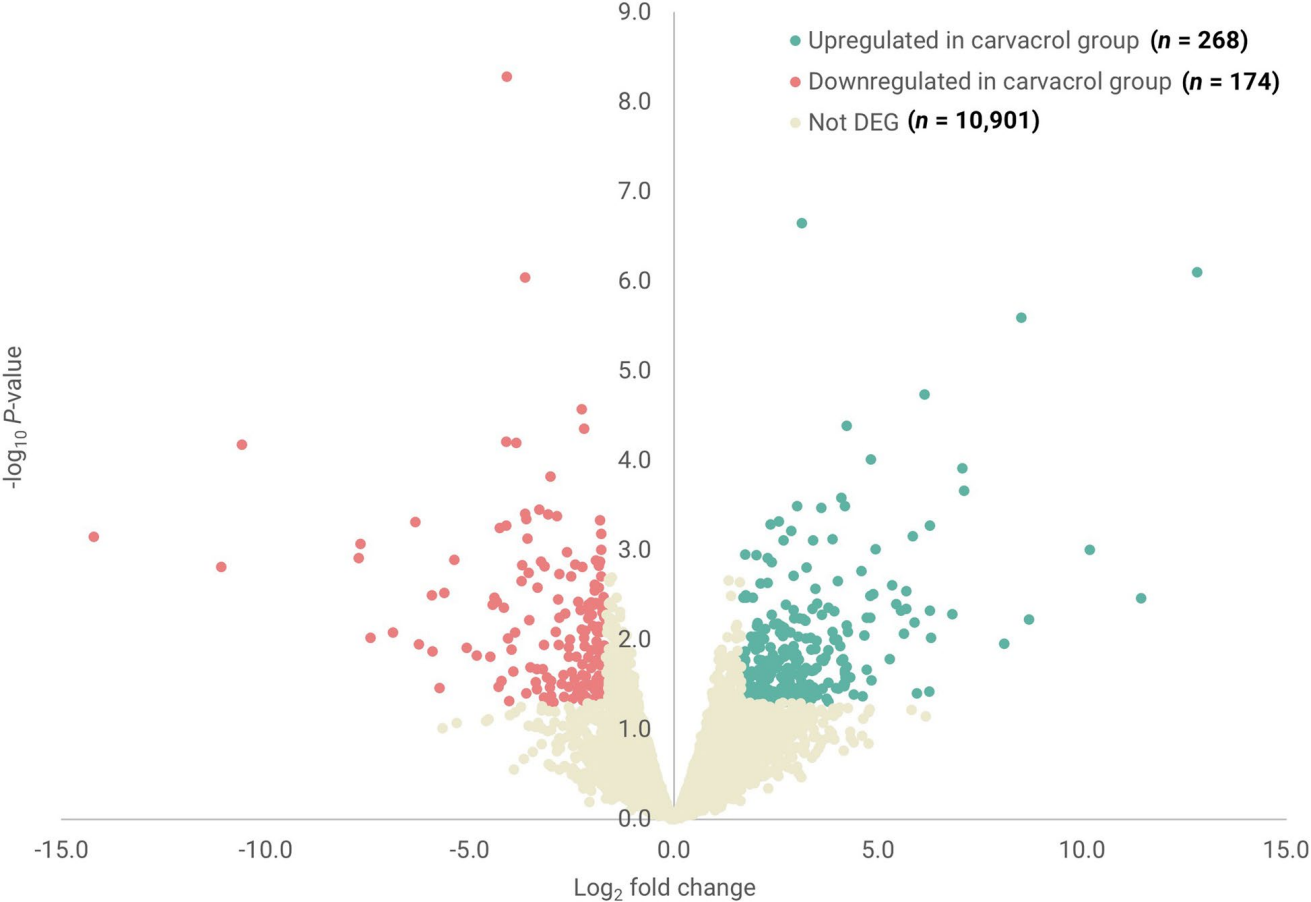
Volcano plot showing the total number of differentially (DEG) and non-differentially (Not DEG) expressed genes in the yolk sac (YS) of broiler chicken embryos at embryonic day 19.5, after in ovo delivery of carvacrol at embryonic day 17.5 compared to the control (saline injected) group. Transcriptomes of 11 samples were used (control; *n* = 5, carvacrol; *n* = 6). Every dot represents one gene. In total, 10,901 genes were found expressed in the YS, from which 442 were DEG (*P* < 0.05, logFC < −0.5 or logFC > 0.5) between the 2 treatments (268 upregulated and 174 downregulated in the carvacrol group compared to the control)

The RNA sequencing (RNAseq) analysis identified transcripts from 10,901 genes in the YS of embryos at E19.5 (Fig. 2 and Additional file 1). The YS transcriptome comparisons between the carvacrol injected and control (saline-injected) groups resulted in the identification of 14 DEG with a false discovery rate (FDR) below 0.10. From these DEG, seven were upregulated and seven were downregulated (Table S2).

**Fig. 3:**
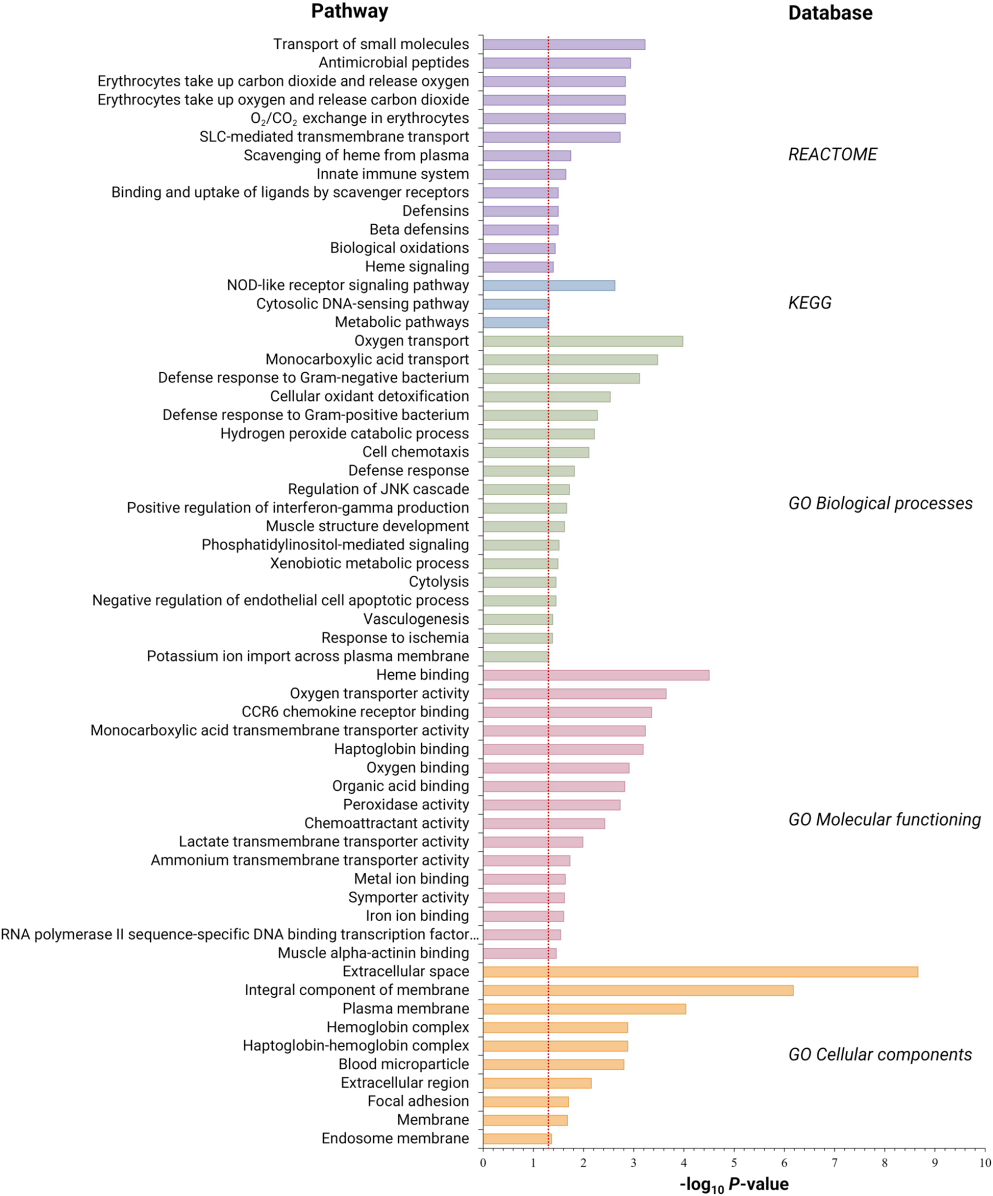
REACTOME, Kyoto Encyclopedia of Genes and Genomes (KEGG) and Gene Ontology (GO) enrichment outputs. GO outputs are divided into Biological processes, Molecular functioning, and Cellular components. Figure shows pathways that were enriched by differentially expressed genes (DEG) in the yolk sac of broiler chicken embryos at embryonic day 19.5, after in ovo delivery of carvacrol at embryonic day 17.5 compared to the control (saline) group. The red line shows the threshold of *P* < 0.05

To allow for pathway analyses, a less stringent cutoff of *P* < 0.05 and logFC < −0.5 or logFC > 0.5 was used instead of an FDR of < 0.10, which resulted in the identification of 442 DEG. From these DEG, 268 genes were upregulated and 174 downregulated in the carvacrol-injected group (*P* < 0.05 and logFC < −0.5 or logFC > 0.5). Functional analyses using REACTOME, KEGG and GO showed that the DEG between the carvacrol injected and control groups resulted in multiple enriched biological pathways (*P* < 0.05) (Fig. 3). Further analyses showed a highly significant enrichment of *P* < 0.01 of immune-related pathways. These included ‘Nucleotide-binding and oligomerization domain (NOD)-like receptor signaling’ (*P* = 0.002), ‘Antimicrobial peptides’ (*P* = 0.001), ‘CCR6 chemokine receptor binding’ (*P* < 0.001), ‘Defense response to Gram-negative bacterium’ (*P* = 0.001), ‘Chemoattractant activity’ (*P* = 0.004), ‘Defense response to Gram-positive bacterium’ (*P* = 0.005), and ‘Cell chemotaxis’ (*P* = 0.008) (Table 1).

**Table 1:** Immunomodulatory pathways^a^ (*P* < 0.01) enriched by in ovo delivery of carvacrol in the yolk sac^b^

Database	Pathway terms	*P*-value	% enriched	Fold enrichment	DEG enriching the pathways	
					Upregulated by carvacrol (*P* < 0.05)	Downregulated by carvacrol (*P* < 0.05)
KEGG	*NOD-like receptor signaling*	0.002	2.56	3.14	*TRPM2*, *CATH1*, *IL18*, *TMEM173*, *DEFB4A*, *PLCB2*, *IKBKE*, *CASP18*, *CATH2*	*CCL5*, *NEK7*
REACTOME	*Antimicrobial peptides*	0.001	1.40	7.04	*CATH1*, *AvBD6*, *AvBD1*, *RSFR*, *AvBD7*, *CATH2*	none
GO	*CCR6 chemokine receptor binding*	0.000	1.16	13.04	*AvBD10*, *AvBD6*, *AvBD1*, *DEFB4A*, *AvBD7*	none
	*Defense response to Gram-negative bacterium*	0.001	1.40	8.02	*CATH1*, *AvBD10*, *RSFR*, *AvBD7*, *CATH2*, *AQP1*	none
	*Chemoattractant activity*	0.004	1.16	7.60	*COLEC10*, *AvBD6*, *AvBD1*, *DEFB4A*, *AvBD7*	none
	*Defense response to Gram-positive bacterium*	0.005	1.16	6.94	*CATH1*, *AvBD10*, *RSFR*, *LYG2*, *CATH2*	none
	*Cell chemotaxis*	0.008	1.16	6.22	*CX3CR1*, *AvBD6*, *AvBD1*, *DEFB4A*, *AvBD7*	none

*AQP1* Aquaporin 1, *AvBD1* Avian beta-defensin 1, *AvBD6* Avian beta-defensin 6, *AvBD7* Avian beta-defensin 7, *AvBD10* Avian beta-defensin 10, *CASP18* Initiator caspase, *CATH1* Cathelicidin-1, *CATH2* Cathelicidin-2, *CCL5* C-C motif chemokine ligand, *COLEC10* Collectin subfamily member 10, *CX3CR1* C-X3-C motif chemokine receptor 1, *DEFB4A* Defensin beta 4 A, *IKBKE* Inhibitor of nuclear factor kappa B kinase subunit epsilon, *IL18* Interleukin 18, *LYG2* Lysozyme g2, *NEK7* NIMA related kinase 7, *PLCB2* Phospholipase C beta 2, *RSFR* Leukocyte ribonuclease A-2, *TMEM173* Transmembrane protein 173, *TRPM2* Transient receptor potential cation channel subfamily M member 2

^a^Immunomodulatory pathways compared to the control (saline) group (*P* < 0.01), including the differentially expressed genes (DEG) responsible for enrichment. Databases used are Kyoto Encyclopedia of Genes and Genomes (KEGG), Gene Ontology (GO), and REACTOME

^b^The yolk sac of broiler embryos at embryonic day 19.5

**Fig. 4:**
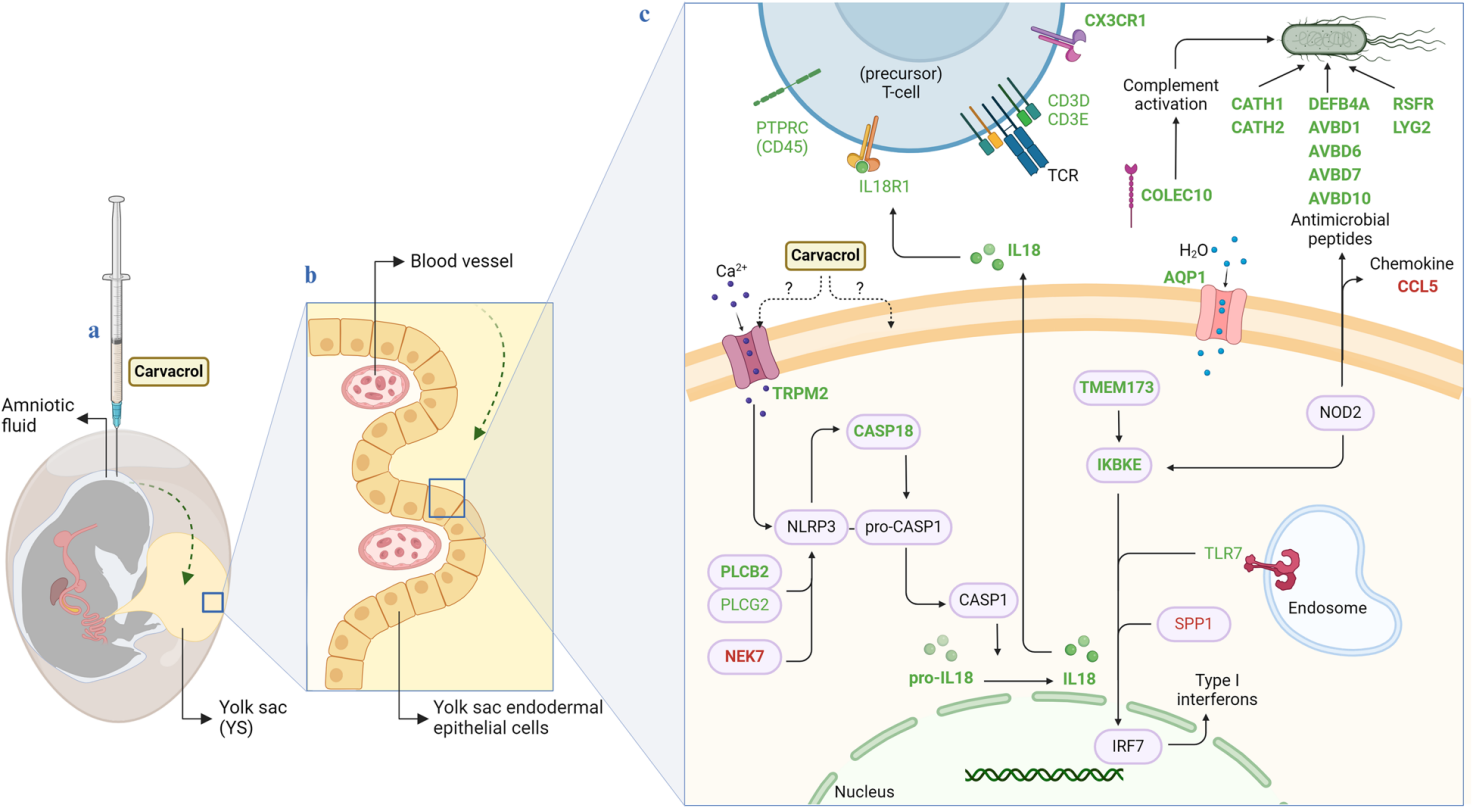
Integration of genes enriching metabolic pathways across the databases Kyoto Encyclopedia of Genes and Genomes (KEGG), Gene Ontology (GO), and REACTOME, to comprehend the functional implications of differentially expressed genes (DEG) identified in the yolk sac (YS) of broiler chickens at embryonic day 19.5 after in ovo delivery of carvacrol at embryonic day 17.5 compared to the control (saline-injected) group. (**a**) After in ovo delivery, carvacrol mainly migrates to the yolk [19]; (**b**) which has a villi-like folded structure with endodermal epithelial cells surrounding blood vessels [6]; (**c**) in the YS, carvacrol enriches immunomodulatory pathways that result in increased expressed antimicrobial defence pathways, as well as an increase in expressed chemotactic pathways. Several upregulated DEG can normally be found on lymphoid cells, such as T-cells, highlighting a possible presence and involvement of (precursor) T-cells in the YS. Bold-printed genes are DEG enriching pathways, while normal printed genes are DEG, but not found to be enriching pathways. Green; upregulated by carvacrol, Red; downregulated by carvacrol. Genes printed in black were not found as DEG but are added to better understand pathway functions. Abbreviations: *AQP1* Aquaporin 1; *AvBD1* Avian beta-defensin 1; *AvBD6* Avian beta-defensin 6; *AvBD7* Avian beta-defensin 7; *AvBD10* Avian beta-defensin 10; *CASP18* Initiator caspase; *CATH1* Cathelicidin-1; *CATH2* Cathelicidin-2; *CCL5* C-C motif chemokine ligand; *CD3D* CD3δ molecule; *CD3E* CD3ε molecule; COLEC10 Collectin subfamily member 10; *CX3CR1* C-X3-C motif chemokine receptor 1; *DEFB4A* Defensin beta 4 A; *IKBKE* Inhibitor of nuclear factor kappa B kinase subunit epsilon; *IL18* Interleukin 18; *IL18R1* Interleukin 18 receptor 1; *LYG2* Lysozyme g2; *NEK7* NIMA related kinase 7; *PLCB2* Phospholipase C beta 2; *PLCG2* Phospholipase C gamma 2; *PTPRC* Protein tyrosine phosphatase, receptor type C; *RSFR* Leukocyte ribonuclease A-2; *SPP1* = Secreted phosphoprotein; *TLR7* Toll-like receptor 7; *TMEM173* Transmembrane protein 173; *TRPM2* Transient receptor potential cation channel subfamily M member 2. Created with BioRender.com

In the ‘NOD-like receptor signaling’ pathway, 11 DEG were involved, of which *TRPM2* (transient receptor potential cation channel subfamily M member 2), *CATH1* (cathelicidin-1), *CATH2* (cathelicidin-2), *IL18* (interleukin 18), *TMEM173* (transmembrane protein 173), *DEFB4A* (defensin beta 4 A), *PLCB2* (phospholipase C beta 2), *IKBKE* (inhibitor of nuclear factor kappa B kinase subunit epsilon) and *CASP18* (initiator caspase) were upregulated by in ovo carvacrol delivery, and *CCL5* (C-C motif chemokine ligand) and *NEK7* (NIMA related kinase 7) were downregulated. In the ‘Antimicrobial peptides’ pathway, six DEG were involved and upregulated, including *CATH1*, *CATH2*, *AvBD1* (avian beta-defensin 1), *AvBD6* (avian beta-defensin 6), *AvBD7* (avian beta-defensin 7) and *RSFR* (leukocyte ribonuclease A-2). For the ‘CCR6 chemokine receptor binding’ pathway, five DEG were involved and upregulated, including *AvBD1*, *AvBD6*, *AvBD7*, *AvBD10* and *DEFB4A*. For the ‘Defense response to Gram-negative bacterium’ pathway, six DEG were involved and upregulated, including *CATH1*, *CATH2*, *AvBD7*, *AvBD10*, *RSFR*, and *AQP1* (aquaporin 1). For the ‘Chemoattractant activity’ pathway, five DEG were involved and upregulated, including *COLEC10* (collectin subfamily member 10), *AvBD1*, *AvBD6*, *AvBD7* and *DEFB4A*. For the ‘Defense response to Gram-positive bacterium’ pathway, five DEG were involved and upregulated, including *CATH1*, *CATH2*, *AvBD10* (avian beta-defensin 10), *RSFR* and *LYG2* (lysozyme g2). Finally, for the ‘Cell chemotaxis’ pathway five DEG were involved and upregulated, including *CX3CR1* (C-X3-C motif chemokine receptor 1), *AvBD1*, *AvBD6*, *AvBD7*, and *DEFB4A*. The genes *CATH1*, *CATH2*, *DEFB4A*, *AvBD1*, *AvBD6*, *AvBD7*, *AvBD10* and *RSFR*, which are all related to antimicrobial peptides, were overlapping between the enriched pathways. All DEG in these pathways are shown in Table 2 and integrated in Fig. 4.

**Table 2:** Differentially expressed genes^a^ in immunomodulatory pathways in the yolk sac^b^ affected by in ovo carvacrol delivery

Gene	Log fold change	*P*-value	Expression^c^ in control group (saline)	Expression in carvacrol group
*AQP1*	0.72	0.021	1,131 ± 242	2,053 ± 258
*AvBD1*	0.83	0.021	3,821 ± 712	7,550 ± 1,558
*AvBD10*	1.18	0.010	1,273 ± 236	3,244 ± 813
*AvBD6*	1.21	0.002	265 ± 31	678 ± 164
*AvBD7*	0.82	0.033	1,743 ± 473	3,424 ± 671
*CASP18*	0.60	0.048	33 ± 5	54 ± 6
*CATH1*	0.95	0.024	314 ± 56	674 ± 183
*CATH2*	0.83	0.038	443 ± 83	883 ± 215
*CCL5*	−2.07	0.008	72 ± 45	19 ± 4
*COLEC10*	0.56	0.050	435 ± 89	708 ± 81
*CX3CR1*	1.27	0.020	5 ± 2	12 ± 3
*DEFB4A*	0.82	0.027	2,435 ± 498	4,755 ± 988
*IKBKE*	0.55	0.027	99 ± 16	160 ± 12
*IL18*	1.01	0.013	86 ± 18	198 ± 46
*LYG2*	0.87	0.001	1,566 ± 277	3,187 ± 399
*NEK7*	−0.60	0.046	2,692 ± 557	2,013 ± 295
*PLCB2*	0.55	0.047	90 ± 12	145 ± 20
*RSFR*	0.88	0.012	564 ± 71	1,159 ± 250
*TMEM173*	0.59	0.025	84 ± 3	139 ± 16
*TRPM2*	0.97	0.050	12 ± 3	25 ± 6

*AQP1* Aquaporin 1, *AvBD1* Avian beta-defensin 1, *AvBD6* Avian beta-defensin 6, *AvBD7* Avian beta-defensin 7, *AvBD10* Avian beta-defensin 10, *CASP18* Initiator caspase, *CATH1* Cathelicidin-1, *CATH2* Cathelicidin-2, *CCL5* C-C motif chemokine ligand, *COLEC10* Collectin subfamily member 10, *CX3CR1* C-X3-C motif chemokine receptor 1, *DEFB4A* Defensin beta 4 A, *IKBKE* Inhibitor of nuclear factor kappa B kinase subunit epsilon, *IL18* Interleukin 18, *LYG2* Lysozyme g2, *NEK7* NIMA related kinase 7, *PLCB2* Phospholipase C beta 2, *RSFR* Leukocyte ribonuclease A-2, *TMEM173* Transmembrane protein 173, *TRPM2* Transient receptor potential cation channel subfamily M member 2

^a^Genes involved in immunomodulatory pathways found in Table 1

^b^Yolk sac of broiler chicken embryos at embryonic day 19.5

^c^Gene expression is shown as Fragments Per Kilobase per Millon mapped reads (Mean ± SE)

Seven other DEG (*P* < 0.05) of relevance to immune functioning, but not enriching any of the previous mentioned pathways, were found and are shown in Table 3. Six of them were upregulated by in ovo delivery of carvacrol (*CD3D*, CD3δ molecule; *CD3E*, CD3ε molecule; *IL18R1*, interleukin 18 receptor 1; *PLCG2*, phospholipase C gamma 2; *PTPRC*, protein tyrosine phosphatase, receptor type C; *TLR7*, Toll-like receptor 7), and one was downregulated (*SPP1*, Secreted Phosphoprotein).

**Table 3:** Differentially expressed immune-related genes in the yolk sac^a^ not involved in pathways of Table 1^b^

Gene	Log fold change	*P*-value	Expression^c^ in control group (saline)	Expression in carvacrol group
*CD3D*	0.759	0.020	60 ± 9	114 ± 20
*CD3E*	0.925	0.006	80 ± 13	169 ± 21
*IL18R1*	0.672	0.040	28 ± 6	50 ± 6
*PLCG2*	0.765	0.042	72 ± 14	133 ± 28
*PTPRC*	0.512	0.045	204 ± 19	324 ± 47
*SPP1*	−2.236	0.009	1,171 ± 704	271 ± 105
*TLR7*	1.051	0.010	42 ± 9	96 ± 18

*CD3D* CD3δ molecule, *CD3E* CD3ε molecule, *IL18R1* Interleukin 18 receptor 1, *PLCG2* Phospholipase C gamma 2, *PTPRC* Protein tyrosine phosphatase, receptor type C, *SPP1* Secreted phosphoprotein, *TLR7* Toll-like receptor 7

^a^Yolk sac of broiler chicken embryos at embryonic day 19.5

^b^Genes were not differentially expressed and are relevant to the current study, but did not enrich pathways in Table 1

^c^Gene expression is showed as Fragments Per Kilobase per Millon mapped reads (mean ± SE)

## Discussion

The aim of this experiment was to study the impact of in ovo delivery of carvacrol on the YS immune defense mechanisms in broiler chickens. Comparing the YS transcriptomes of the carvacrol-injected to the saline-injected chickens resulted in the identification of 442 DEG (268 upregulated and 174 downregulated for in ovo carvacrol delivery compared to the control). The list of DEG significantly enriched several pathways associated with immune response such as the upregulation of the ‘NOD-like receptor signaling’ which involves receptors that respond to non-self-components [24]. Other enriched pathways relevant to defense mechanisms triggered by NOD-like receptor signaling included the ‘Antimicrobial peptides’, ‘Defense response to Gram-negative bacterium’ and ‘Defense response to Gram-positive bacterium’ pathways as well as ‘CCR6 chemokine receptor binding’, ‘Chemoattractant activity’ and ‘Cell chemotaxis’ pathways. Almost all of the DEG involved in these pathways were upregulated by in ovo delivery of carvacrol (i.e., *AQP1*, *AvBD1*, *AvBD6*, *AvBD7*, *AvBD10*, *CASP18*, *CATH1*, *CATH2*, *COLEC10*, *CX3CR1*, *DEFB4A*, *IKBKE*, *IL18*, *LYG2*, *PLCB2*, *RSFR*, *TMEM173* and *TRPM2*), except for *CCL5* and *NEK7*.

Based on pathway enrichment and involved DEG’s, it seems that several immunomodulatory mechanisms in the embryonic YS are influenced by in ovo carvacrol delivery. In ovo delivery of carvacrol triggered an increased expression of antimicrobial peptides and specifically avian beta-defensins, which constitute an important mechanism in innate immune defences and are amongst the molecules produced after NOD-like receptor pathway activation [25]. Upregulation of these genes also resulted in enrichment of pathways related to antimicrobial defence. Antimicrobial peptides are being considered a potential alternative for antibiotics, by playing an important role in innate immune defence by interacting with bacterial membranes [26, 27]. Initially they have the capability to bind to bacteria, subsequently penetrating their membranes resulting in bacterial lysis and cell death [26]. This, in turn, indirectly further modulates immune responses by activation of the recruitment of leukocytes [27–29]. Due to these characteristics, antimicrobial peptides are being considered as a promising alternative to antibiotics, not only due their efficacy but also because the demonstrate lower susceptibility to the development of antimicrobial resistance [29]. Several antimicrobial peptides can be found in the embryonic YS [6], including avian beta-defensins, which are specific to avian species. It was shown that intra-amniotic in ovo delivery of antimicrobial peptides, and specifically AvBD1 and CATH2, offered protection against YS and respiratory infections caused by *Escherichia coli* [29, 30]. Furthermore, starting at the end of incubation the yolk contents are transported into the gastrointestinal tract [31], providing a potential delivery route of antimicrobial peptides to the developing gastrointestinal tract which agrees with speculations that avian beta-defensins would be able to protect the developing embryo [6]. The upregulation of the expression of antimicrobial peptides, such as the cathelicidins CATH1, CATH2 as well as the avian defensins AvBD1, AvBD6, AvBD7 and AvBD10 and beta-defensin DEFB4A, also known as AvBD2 (avian beta-defensin 2) [32] occurred most likely through the NOD2 signaling constitutive of the ‘*NOD-like receptor signaling*’ pathway as shown by a link in DEG through database KEGG.

Antimicrobial peptides also have immunomodulatory effects by playing a role in chemotaxis, which is the recruitment of cells in response to a stimulus. This view was supported by significant enrichment of at least three pathways including ‘CCR6 chemokine receptor binding’, ‘Chemoattractant activity’ and ‘Cell chemotaxis’ [29]. Chemotactic factors promote recruitment of immune cells to sites of inflammation, but can also aid in adaptive immune development, anti-inflammatory function by attracting leukocytes and can aid in wound healing [27]. Upregulated chemotactic factors included COLEC10, which is a soluble pattern recognition receptor that can identify pathogens and can result in enhanced pathogen clearing through complement activation [33, 34], and CX3CR1, a chemokine receptor found on lymphocytes [35]. However, the chemokine CCL5, which attracts effector cells to sites of inflammation was found to be downregulated [36]. Next to CX3CR1 and IL18R1, other receptors that can be found on lymphocytes, such as PTPRC (also known as CD45) [37], and the combination of CD3D and CD3E which together make up a part of the T-cell receptor [38], were found to be upregulated. Apparently, T-cells or their precursors may already have a functional role in the embryonic YS, which aligns with the finding that lymphoid progenitors are present in the YS [39]. The finding that both antimicrobial and chemotactic pathways were enriched in the YS by in ovo delivery of carvacrol, could also indicate that carvacrol was perceived as a foreign molecule that needed to be cleared. This is consistent with previous research showing the enrichment of xenobiotic detoxification pathways in the jejunum at hatch after in ovo delivery of oregano essential oil (containing 74% of carvacrol) in a dose-dependent manner [21]. In the same experiment, it was shown that amounts of up to 10 µL of the essential oil did not impact hatchability. In the current study 5 µL of carvacrol was used, so while carvacrol may have provoked immune responses, the used dosage was expected to be safe.

Another outcome of the transcriptomic analysis was that carvacrol has the potential to improve in ovo vaccination for Marek’s disease through the upregulation of IL18. In brief, TRPM2, PLCB2 and PLGG2 have the potential to stimulate inflammatory pathways through Ca^2+^ dependent NLRP3 (NOD-like receptor 3) [40] signaling which activates CASP18, a protease member of the caspase-family, with a major role in inflammation [41] and leads to the formation of CASP1 (caspase 1) through pro-CASP1 formation. Importantly, activated CASP1 cleaves pro-IL18 into IL18 [42]. The cytokine IL18 plays a key role in inducing Th1 immune responses by binding to IL18R1 (another upregulated DEG in this study) found on T-cells [43]. IL18 is a potent adjuvant for Newcastle disease and infectious bursal disease vaccine, indicating that the carvacrol-induced upregulation of IL-18R may have the potential to improve in ovo vaccination for Marek’s disease [44, 45].

The specific mechanisms by which carvacrol influenced the activation of immune signaling pathways is unknown. It could be speculated that carvacrol being a small, lipophilic molecule has the capacity to freely cross eukaryotic cell membranes and interact with transcription factors, as shown in previous research using oregano essential oil [21]. In contrast, feeding carvacrol post-hatch under viral or bacterial challenges resulted in downregulation of the TLR/NF-κB transcription pathway [15, 18]. The different results might be related to the fact that the results presented here were under neutral conditions. In addition, carvacrol is known to activate TRPA1 and TRPV3 ion channels [46, 47]. Moreover, the TRP channel TRPM2, which is thought to be downregulated by carvacrol [48], was upregulated in the current study. There could be several explanations for the observed differences between the current study and other research; (a) most studies focus on mammals, whereas it is known that several immune mechanisms in birds differ, such as in the function of lymphoid organs, the presence of heterophils instead of neutrophils, and variations in antibodies and immune receptors [49], (b) mechanisms during embryonic development can vary from those in the post-hatching phase, (c) mechanisms may work differently in the yolk sac. TRPM2 is a channel known to facilitate Ca^2+^ influx [50]. Since carvacrol is known to increase intracellular Ca^2+^ concentrations [51], this could be explained by this increase in TRPM2 expression in the YS.

The pro-inflammatory effects observed following in ovo delivery of carvacrol are compatible with previous results involving plant-derived compounds stimulating the NOD-like receptor signaling pathways amongst others. For example, the immune response to a Newcastle disease vaccine was boosted partially through the NOD-like receptor pathway when ginseng extract was used as part of the adjuvant of the vaccine [52]. Some of these effects involving the NOD-like receptor pathway were also observed in chicken embryos (in ovo). Kong et al. [53], injected glycerol monolaurate (GML) on E17.5 showing improved intestinal morphology and antioxidant status in an LPS-challenged broiler embryo model. Inflammation, while critical for pathogen defence, can lead to tissue damage and developmental complications when excessive. Thus, further investigation around the potential effect of in ovo carvacrol (and other functional substances) on the developing immune system may be required. In particular, it will be crucial determining precisely safe doses and timing of administration of functional ingredients that may foster immune development while minimizing the risk of harmful inflammatory responses.

While in ovo delivery of carvacrol could prepare the newly hatched chicken against bacterial pathogens by promoting antimicrobial peptide production in the YS, this results also flagged a potential threat. The enriched pathways indicate that carvacrol may elicit a pro-inflammatory immune response, potentially because it is seen as a foreign body. Thus, this indicates the importance of the right dose to be injected. Moreover, the current study has focused on gene expression, and whether this translated to production of effector molecules remains unknown and warrants further research.

## Conclusions

In conclusion, in ovo delivery of carvacrol may stimulate a detoxification response while showing the potential to enhance the expression of antimicrobial peptides in the YS. These insights contribute to our understanding of potential strategies for enhancing innate immune defenses in early poultry development and show the potential of in ovo delivery of carvacrol as an alternative to decrease the dependence on antibiotic treatments particularly in the early post-hatching phase. Combining in ovo delivery of carvacrol with an experimental pathogenic challenge model could give more insights into these findings.

## Supplementary Information

Additional file 1: Supplementary methods. Table S1 Target genes and primer sequences of immune mediators used for RT-qPCR analysis. Fig. S1 Correlation between gene expression level of 6 candidate genes, using RNAseq and qPCR. Table S2 Differentially expressed genes in the yolk sac of broiler embryos at embryonic day 19.5, affected by in ovo delivery of carvacrol with a false discovery rate (FDR) of < 0.10.

## Data Availability

The datasets used and/or analysed during the current study are available from the corresponding author on reasonable request.

## Abbreviations

AQP1: Aquaporin 1
AvBD: Avian beta-defensin
AvBD1: Avian beta-defensin 1
AvBD10: Avian beta-defensin 10
AvBD6: Avian beta-defensin 6
AvBD7: Avian beta-defensin 7
CASP18: Initiator caspase
CATH1: Cathelicidin-1
CATH2: Cathelicidin-2
CCL5: C-C motif chemokine ligand
CD3D: CD3δ molecule
CD3E: CD3ε molecule
COLEC10: Collectin subfamily member 10
CX3CR1: C-X3-C motif chemokine receptor 1
DAVID: Database for Annotation, Visualization and Integrated Discovery
DEFB4A: Defensin beta 4A
DEG: Differentially expressed genes
FC: Fold change
FDR: False discovery rate
GLM: General linear model
GO: Gene Ontology
IKBKE: Inhibitor of nuclear factor kappa B kinase subunit epsilon
IL18: Interleukin 18
IL18R1: Interleukin 18 receptor 1
KEGG: Kyoto Encyclopedia of Genes and Genomes
LYG2: Lysozyme g2
NEK7: NIMA related kinase 7
NOD: Nucleotide oligomerization domain
PBS: Phosphate buffered saline
PLCB2: Phospholipase C beta 2
PLCG2: Phospholipase C gamma 2
PTPRC: Protein tyrosine phosphatase, receptor type C
RIN: RNA integrity number
RNAseq: RNA sequencing
RSFR: Leukocyte ribonuclease A-2
RT-qPCR: Reverse transcription quantitative real-time polymerase chain reaction
SPP1: Secreted Phosphoprotein
TLR7: Toll-like receptor 7
TMEM173: Transmembrane protein 173
TRPM2: Transient receptor potential cation channel subfamily M member 2
YS: Yolk sac
