# Supplementary material for: In ovo delivery of carvacrol triggers expression of chemotactic factors, antimicrobial peptides and pro-inflammatory pathways in the yolk sac of broiler chicken embryos
Source: J Anim Sci Biotechnol. 2025 Jan 19;16:8. doi: 10.1186/s40104-024-01131-3 (PMC11742807; doi:10.1186/s40104-024-01131-3)
Supplement: Supplementary file 1 — Additional file 1: Supplementary methods. Table S1 Target genes and primer sequences of immune mediators used for RT-qPCR analysis. Fig. S1 Correlation between gene expression level of 6 candidate genes, using RNAseq and qPCR. Table S2 Differentially expressed genes in the yolk sac of broiler embryos at embryonic day 19.5, affected by in ovo delivery of carvacrol with a false discovery rate (FDR) of < 0.10. [file 40104_2024_1131_MOESM1_ESM.docx]

**Additional file 1**

**Supplementary methods**

***Methods for RT-qPCR validation***

Total RNA was isolated from yolk sac tissue, using RNeasy Universal kit (Qiagen, Hilden, Germany). RNA concentrations were determined by Nanodrop 1000 (Thermo Scientific, Waltham, MA, USA). Total RNA from each sample was reverse transcribed into cDNA, using a cDNA synthesis kit (Qiagen, Hilden, Germany). cDNA was verified by 1.5% agarose gel electrophoresis.

For qRT-PCR, 1 µL cDNA and 1 µL forward and reverse primers (4 nmol/L) were added to 5 µL SYBR green-based mix (QuantiNova, Qiagen, Hilden, Germany) and filled up with 3 µL RNAse free water to a total volume of 10 µL. The primer sequences (Sigma Aldrich, St. Louis, MO, USA) used for real-time PCR are listed in Table S1. RT-PCR was carried out on a Viia7 real-time PCR machine (Thermo Scientific, Waltham, MA, USA). All reactions were analysed in duplicate. PCR was performed under the following conditions: 2 min 95 °C, followed by 45 cycles of 5 s 95 °C and 20 s 60 °C. A melt curve was produced at the end of the run to determine single product amplification.

In SAS 9.4, the PROC CORR procedure was used to correlate log-transformed qRT-PCR data (Ct-values) with log-transformed RNAseq data (RPKM).

**Table S1** Target genes and primer sequences of immune mediators used for RT-qPCR analysis

| **Gene** | **Accession No.** | **Primer sequence (5'→3')^1^** | **Reference** |
| --- | --- | --- | --- |
| *BACT* | X00182 | F: CAACACAGTGCTGTCTGGTGGTA | (St. Paul et al., 2011) |
|  |  | R: ATCGTACTCCTGCTTGCTGATCC |  |
| *CD3D* | NM_205512.2 | F: TGTTGTCGCCACTGTCTTGCTG | (Song et al., 2021) |
|  |  | R: GTCCATCATTCCGCTCACCAAGG |  |
| *IL8L1* | NM_205018.2 | F: CACGTTCAGCGATTGAACTC | (Santos et al., 2019) |
|  |  | R: GACTTCCACATTCTTGCAGTG |  |
| *NFKB* | XM_046915553.1 | F: GAAGGAATCGTACCGGGAACA | (Chiang et al., 2009) |
|  |  | R: CTCAGAGGGCCTTGTGACAGTAA |  |
| *TGFB4* | M31160 | F: ACCTCGACACCGACTACTGCTT | (Lammers et al., 2010) |
|  |  | R: ATCCTTGCGGAAGTCGATGT |  |
| *TNFA* | XM_046927265.1 | F: CCCCTACCCTGTCCCACAA | (Liu et al., 2019) |
|  |  | R: TGAGTACTGCGGAGGGTTCAT |  |

*BACT* β-Actin, *CD3D* CD3 δ subunit of T-cell Receptor Complex, *IL8L1* Interleukin 8, *NFKB* Nuclear factor κB, *TGFB4* Transforming growth factor β, *TNFA* Tumor necrosis factor α

^1^F: forward primer; R reverse primer


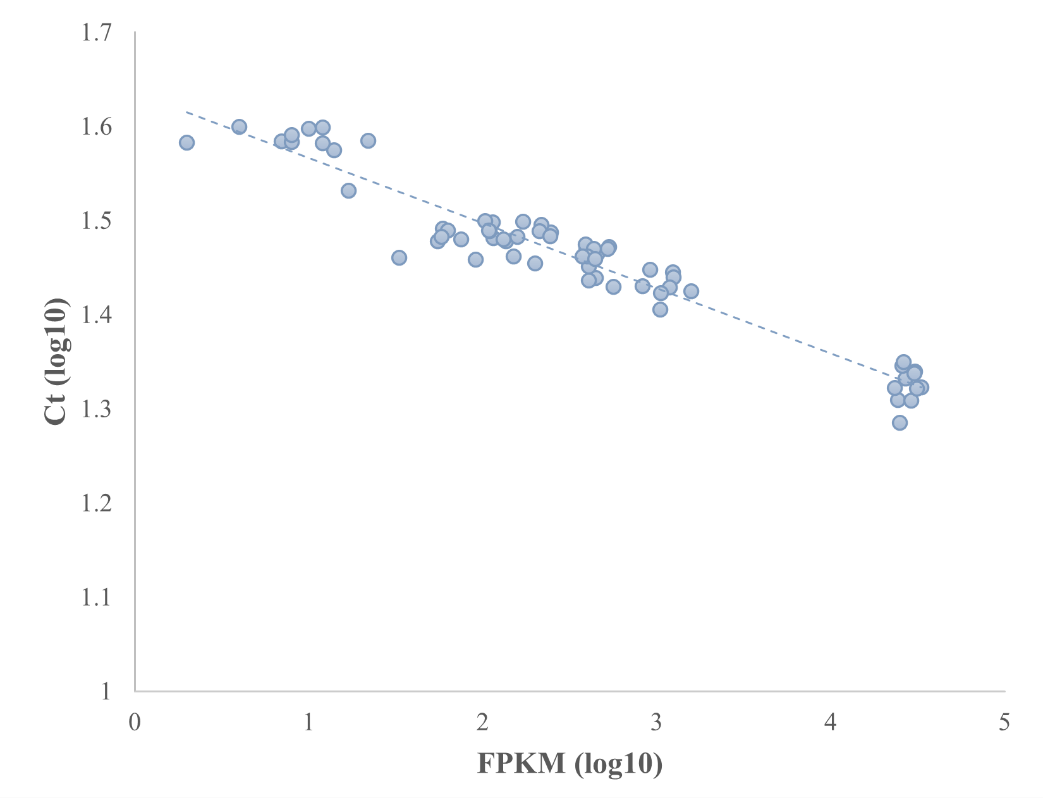


**Fig. S1** Correlation between gene expression level of 6 candidate genes, using RNAseq and qPCR. Ct: CT value; FPKM: Fragments Per Kilobase of transcript per Million mapped reads. The results showed a high correlation coefficient (-0.93; *P* < 0.0001) between gene expression of these genes measured using qPCR (Ct value) and RNAseq (RPKM)

**Supplementary results**

**Table S2** Differentially expressed genes in the yolk sac of broiler embryos at embryonic day 19.5, affected by in ovo delivery of carvacrol with a false discovery rate (FDR) of < 0.10

| **Gene** | **Log Fold Change** | **FDR** | **Expression in control group (saline)** | **Expression in carvacrol group** |
| --- | --- | --- | --- | --- |
| *ATP6V0C* | -0.68 | 0.041 | 7368 ± 482 | 5171 ± 546 |
| *C2CD3* | 1.27 | 0.053 | 106 ± 16 | 286 ± 55 |
| *DIO3* | -3.18 | 0.061 | 19 ± 8 | 2 ± 1 |
| *DSEL* | 0.95 | 0.001 | 1190 ± 57 | 2496 ± 137 |
| *ENPP7* | 1.85 | 0.033 | 59 ± 11 | 228 ± 48 |
| *FJX1* | 1.45 | 0.081 | 11 ± 2 | 33 ± 6 |
| *FNIP1* | -1.10 | 0.002 | 502 ± 56 | 260 ± 30 |
| *HBE1* | 2.13 | 0.095 | 87 ± 22 | 396 ± 108 |
| *KHDRBS3* | -1.16 | 0.061 | 93 ± 12 | 46 ± 7 |
| *KLF5* | -1.23 | 0.061 | 570 ± 120 | 276 ± 35 |
| *OSCP1* | 3.86 | 0.002 | 13 ± 1 | 200 ± 60 |
| *TMEM38A* | -0.66 | 0.053 | 2144 ± 180 | 1493 ± 118 |
| *TOPAZ1* | 2.56 | 0.006 | 2 ± 1 | 11 ± 1 |
| *ZFPM1* | -1.23 | 0.000 | 433 ± 51 | 205 ± 15 |

*ATP6V0C* ATPase H^+^ transporting V0 subunit c, *C2CD3* C2 domain containing 3 centriole elongation regulator, *DIO3* Deiodinase, iodothyronine type III, *DSEL* Dermatan sulfate epimerase like, *ENPP7* Ectonucleotide pyrophosphatase/phosphodiesterase 7, *FJX1* Four jointed box 1, *FNIP1* Folliculin interacting protein 1, *HBE1* Hemoglobin subunit epsilon 1, *KHDRBS3* KH RNA binding domain containing, signal transduction associated 3, *KLF5* Kruppel like factor 5, *OSCP1* Organic solute carrier partner 1, *TMEM38A* Transmembrane protein 38A, *TOPAZ1* Testis and ovary specific PAZ domain containing 1, *ZFPM1* Zinc finger protein, FOG family member 1

Gene expression is showed as Fragments Per Kilobase per Millon mapped reads (Mean ± SE)
